# Structural and Functional Comparisons of Retroviral Envelope Protein *C*-Terminal Domains: Still Much to Learn

**DOI:** 10.3390/v6010284

**Published:** 2014-01-16

**Authors:** Jonathan D. Steckbeck, Anne-Sophie Kuhlmann, Ronald C. Montelaro

**Affiliations:** 1 Microbiology and Molecular Genetics, University of Pittsburgh School of Medicine, Pittsburgh, PA 15261, USA; E-Mails: jds170@pitt.edu (J.D.S.); ask181@pitt.edu (A.-S.K.); 2 Center for Vaccine Research, University of Pittsburgh, Pittsburgh, PA 15261, USA

**Keywords:** retroviruses, HIV, MuLV, JSRV, cytoplasmic domain, *C*-terminal tail

## Abstract

Retroviruses are a family of viruses that cause a broad range of pathologies in animals and humans, from the apparently harmless, long-term genomic insertion of endogenous retroviruses, to tumors induced by the oncogenic retroviruses and acquired immunodeficiency syndrome (AIDS) resulting from human immunodeficiency virus infection. Disease can be the result of diverse mechanisms, including tumorigenesis induced by viral oncogenes or immune destruction, leading to the gradual loss of CD4 T-cells. Of the virally encoded proteins common to all retroviruses, the envelope (Env) displays perhaps the most diverse functionality. Env is primarily responsible for binding the cellular receptor and for effecting the fusion process, with these functions mediated by protein domains localized to the exterior of the virus. The remaining *C*-terminal domain may have the most variable functionality of all retroviral proteins. The *C*-terminal domains from three prototypical retroviruses are discussed, focusing on the different structures and functions, which include fusion activation, tumorigenesis and viral assembly and lifecycle influences. Despite these genetic and functional differences, however, the *C*-terminal domains of these viruses share a common feature in the modulation of Env ectodomain conformation. Despite their differences, perhaps each system still has information to share with the others.

## 1. Introduction

Retroviruses are a diverse family of enveloped RNA viruses that can be broadly categorized into two groups based on genome complexity: the simple retroviruses and the complex retroviruses. All retrovirus genomes contain three major open reading frames that encode the viral structural and enzymatic proteins: *gag*, *pol* and *env.* In addition to the three major genes, an additional domain, *pro*, codes for the viral protease that is also present in all retroviruses. Distinguishing them from simple retroviruses, complex retroviruses also encode a number of accessory proteins that carry out additional virus-specific functions.

The proteins common to all retroviruses (Gag, Pol, Pro and Env) have the same function regardless of the specific virus. Gag, the major structural protein, forms the viral capsid. Pol is the viral polymerase and is responsible for converting the single-stranded viral RNA genome into double-stranded DNA through its reverse transcriptase and RNaseH activities. Pro cleaves the Gag, Gag-Pro and Gag-Pro-Pol polyproteins into their mature forms and, in some viruses, also removes a *C*-terminal peptide from Env. Env is the receptor binding protein, facilitating the early steps in the virus-cell interaction and additionally drives the fusion process between the viral and cellular membranes. In mediating these basic replication functions, the retroviral proteins interact with numerous cellular cofactors that regulate the specificity and levels of protein function at various stages of viral replication. The remainder of this review focuses on the functional properties of the *C*-terminal tail (CTT) of the Env protein, a domain of Env that is common to all retroviruses, but one that varies widely in length and functionality.

Env, the retroviral envelope protein, is the major viral protein present on the surface of retroviral particles. Env is translated as a polyprotein that is subsequently extensively post-translationally modified during trafficking through the biosynthetic pathway (reviewed for HIV in [1]). Briefly, Env is cleaved by a furin-like protease in the Golgi into its two subunits: the surface unit (SU) protein and the transmembrane (TM) protein. These two proteins remain non-covalently associated in most retroviruses and further assemble into a homotrimeric complex that is the active form of Env. Further, Env SU is cotranslationally modified at a relatively large number of *N*-linked and a few *O*-linked glycosylation sites, with the extent of glycosylation dependent on a particular viral Env sequence. The SU amino acid sequence exhibits a high degree of variation, with hypervariable regions interspersed in the primary sequence with relatively conserved regions, with the conserved regions predominantly composing the folding core of the protein, while the variable regions provide the outer surface of SU.

Env TM is composed of three domains: the ectodomain; the membrane-spanning domain (MSD); and the *C*-terminal domain. The ectodomain of TM contains the fusion peptide at its *N*-terminus, which inserts into the cellular membrane post-receptor binding to begin the fusion process. Conformational changes in the ectodomain bring the cell and viral membranes in close contact, which ultimately results in the fusion of the cellular and viral lipid membranes. The primary function of the MSD is to anchor TM in the cellular/viral lipid membrane. While the specifics regarding the sequence and domain size differ for each individual retrovirus, the involved domains, and subdomains, are similar and have (predominantly) similar functions. The remaining domain, the TM *C*-terminal domain, is likely the most variable in terms of size (length) and functionality among the various retroviruses. The remainder of this review will focus on the Env *C*-terminal domain, or cytoplasmic tail, from three well-characterized retroviruses: murine leukemia virus; Jaagsiekte sheep retrovirus; and human immunodeficiency virus (HIV). 

## 2. Retroviral *C*-Terminal Domains

**Figure 1:**
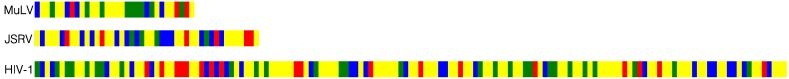
Representative retrovirus *C*-terminal domains. The *C*-terminal domains from Moloney murine leukemia virus (MuLV; 32 residues), Jaagsiekte sheep retrovirus (JSRV; 45 residues) and human immunodeficiency virus (HIV-1; 151 residues) are presented to demonstrate the differences in the lengths of the domains among the different viruses. Each residue is represented by a rectangle colored by the side chain chemical property. Blue: basic; red: acidic; green: polar, not charged; yellow: hydrophobic.

The majority of retroviruses have a short *C*-terminal domain (<50 amino acids) that is localized to the cellular cytoplasm or to the inside of the viral membrane (Figure 1). The lentiviruses, on the other hand, are unique among retroviruses in that they have long (~150 amino acid) *C*-terminal domains. While initially, the lentiviral TM was also thought to be a type I membrane protein [2], its membrane topology has recently become an emerging topic of research and debate [3,4,5,6,7]. Less controversial than the topology of the lentiviral *C*-terminal domain is its extensive functionality (recently reviewed in [1,8,9,10]), a trait that it shares with the *C*-terminal domains of other retroviruses. 

### 2.1. Murine Leukemia Virus (MuLV)

Murine leukemia viruses (MuLV) are a group of viruses that belong to the *Gammaretrovirus* genus of the *Retroviridae* family. MuLV, like Jaagsiekte sheep retrovirus (JSRV), are simple retroviruses and have many exogenous and endogenous species. The particular focus here will be on the exogenous viruses that cause transmissible leukemia in mice, with a prototypical virus being Moloney MuLV, named for its discoverer [11]. MuLV integrates randomly into tissues in the preleukemic stage, but eventually causes tumors through the integration of the viral genome into unique sites of the cellular chromosomal DNA [12].

Like all retroviral Env proteins, the MuLV Env is responsible for receptor binding and membrane fusion. The importance of the cytoplasmic tail of MuLV in viral replication was initially recognized by the identification of a peptide, the R peptide, which is cleaved during Env polyprotein maturation [13].

The R peptide is comprised of the 16 *C*-terminal amino acids from MuLV Env and is cleaved during viral maturation [13]. Initial speculation suggested that R peptide cleavage might play a role in virus assembly [13]. More recent studies have since determined a more precise function for the R peptide. Early studies demonstrated that cleavage of the R peptide from MuLV Env was essential for the cell fusion activity of Env [14,15,16]. The addition of the MuLV cytoplasmic tail, and, specifically, the R peptide sequence, to a truncated simian immunodeficiency virus (SIV) Env protein [17] and influenza virus hemagglutinin protein [18] resulted in the inhibition of fusion activity. Studies that more specifically map the sequences involved in fusion inhibition by the R peptide have determined that truncations greater than seven amino acids from the *C*-terminal end of the R peptide result in increased fusion activity and that a leucine at position 627 plays an important role in fusion inhibition [19]. Other studies have identified other sites in the cytoplasmic tail five or 10 amino acids upstream from the R peptide that can suppress the fusion inhibition exerted by the R peptide, where insertions in these regions led to fusion despite the presence of the R peptide [20]. Finally, a recent study has provided some insight into the mechanism by which the R peptide inhibits fusion. Using cryo-electron microscopy (cryo-EM) of MuLV Env proteins isolated from solubilized viral particles, Garoff and colleagues demonstrated that trimerization of the R peptide sequence serves to hold the legs of TM together in full-length uncleaved MuLV Env, preventing the activation of the TM for fusion. In marked contrast, the TM legs are distinctively separated in mature Env complexes in which the R peptide has been removed by proteolytic cleavage [21]. The modulation of Env ectodomain conformation by changes in the *C*-terminal domains is, as will be seen, an apparently common theme in the retrovirus Env function.

### 2.2. Jaagsiekte Sheep Retrovirus (JSRV)

JSRV is a retrovirus in the *Betaretrovirus* genus that is the causative agent of ovine pulmonary adenocarcinoma (OPA). JSRV is organized as a simple retrovirus, containing the *gag*, *pol* and *env* genes. JSRV also contains an additional open reading frame, ORF-x, of unknown significance, though it remains unclear if ORF-x encodes a functional protein [22,23]. OPA is a contagious disease resulting in tumors that originate in the distal lung and that shares clinical, radiological and histopathological features with a human cancer, bronchioalveolar cancer (BAC). As such, OPA and JSRV are an interesting model system by which to study the virological mechanisms of cancer, with potential applications for understanding human pulmonary carcinomas.

Tumorigenesis of JSRV results from the transformation of differentiated lung epithelial cells in the alveoli and the bronchioli following infection. While the onset of OPA can be rapid under experimental conditions [24], in contrast to the acute oncogenic retroviruses that contain viral oncogenes derived from normal cellular genes, JSRV is not thought to contain a viral oncogene and has no sequence homology with any known cellular oncogene [25]. In addition, the deletion of the extra open reading frame, ORF-x, which would be the likely candidate for an oncogenic sequence, has no effect on cellular transformation *in vitro*, making it unlikely that it functions as a viral oncogene. Since it is clearly established that JSRV is the causative agent of OPA [26], but JSRV contains no sequences with homology to a known oncogene, how then is JSRV causing tumors in the lungs of sheep?

The perhaps surprising answer is that JSRV induces tumor formation through the oncogenic properties of its Env protein. JSRV Env has been shown to transform multiple cell lines *in vitro* (reviewed in [22,23]). Importantly, JSRV Env is oncogenic *in vivo*, inducing lung tumors in mice [27] and sheep [28]. Cell transformation by JSRV Env is known to involve three signaling pathways: (1) the phosphatidylinositol-3 kinase (PI3K)-dependent and -independent Akt pathway; (2) the Raf-MEK-MAPK pathway; and (3) the RON-Hyal2 pathway [29]. It is unclear how JSRV activates both the Akt and the Raf-MEK-MAPK pathways, as no direct interactions between JSRV Env and constituents of those pathways have been observed. For the RON-Hyal2 pathway, JSRV Env binds Hyal2, its entry receptor, which leads to Hyal2 degradation, which frees RON from Hyal2 inhibition, allowing its activation and eventual cellular transformation [30]. 

The TM subunit of the Env protein is the main determinant of cell transformation, with SU also having some effect. In particular, the cytoplasmic tail of TM is essential for the transformation of multiple cell lines [22,23]. This cytoplasmic tail contains a YXXM peptide motif that is a putative binding site for the p85 regulatory subunit of PI3K [31]. However, while the tyrosine residue of the YXXM motif is important for Env-mediated transformation, the motif itself does not appear to be directly involved in binding and activation of PI3K [29]. Recently, an additional function of the JSRV cytoplasmic tail has been determined, where truncations of the tail resulted in increased fusogenicity, which decreased the dependence on the normal low-pH requirement for fusion [32]. The fusion-enhancing truncations were accompanied by conformational changes in the ectodomain of TM, suggesting that the cytoplasmic domain of JSRV plays a regulatory role in the overall Env structure.

### 2.3. Human Immunodeficiency Virus (HIV)

In contrast to JSRV and MuLV, the cytoplasmic domain of the HIV TM protein, gp41, is long, comprising approximately 150 amino acid residues. Results from early topogenesis studies led to the view of gp41 (and, thus, Env as a whole) as a type I membrane protein, with an extracellular *N*-terminus, a single MSD and an approximately 150 amino acid-long cytoplasmic *C*-terminal tail (CTT) [2]. More recent studies, however, indicate that the CTT topology may be more dynamic and complex than previously thought, as most of the CTT sequence can be surface exposed in up to 30% of the Env protein expressed at the cell surface in transfected or infected cells [4]. In contrast, the CTT sequences appear to be exclusively internal in viral particles [4,5].

The presence of a very long CTT is not unique to HIV, as other lentiviruses, such as the simian immunodeficiency virus (SIV) and the equine infectious anemia virus (EIAV), have similarly long CTT sequences, at 150 and 200 amino acids, respectively [33]. The presence of a long CTT in most lentiviruses suggests an important functional role, as viruses do not generally replicate non-functional sequences. This functional importance is supported by the finding that the truncation of the CTT leads to *in vivo* suppression of viral replication in animal models [34]. A decade earlier, however, studies demonstrated that the CTT was dispensable for *in vitro* viral replication [35,36,37], leading to a long-held view that the CTT was not functionally important. This view was subsequently popularized by the finding that the truncation of the CTT led to increased Env incorporation into the virion, which was an important consideration at the time as a means of elevating the anti-Env immune response in experimental vaccines. It is now generally accepted that the CTT plays multiple important functional roles as a determinant of Env structural and functional properties in the virus lifecycle. However, there is very little direct experimental data to characterize the CTT structure; no full-length atomic level structures exist for any CTT sequences.

#### 2.3.1. CTT Structure

**Figure 2:**
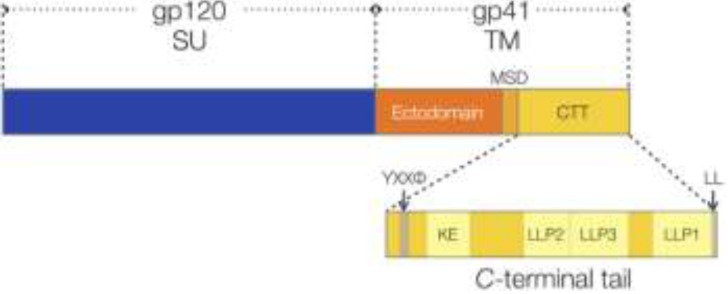
Schematic of HIV Env. The HIV Env is presented, with a focus on the *C*-terminal tail (CTT). The immune reactive Kennedy epitope (KE) and the lentivirus lytic peptides (LLPs) are presented, along with the functional endocytic motifs (YXXϕ and LL). SU, surface unit; TM, transmembrane; MSD: membrane-spanning domain.

Some CTT sequences have been studied structurally using peptide analogs and site-directed mutations. The most well-studied domains, the lentivirus lytic peptides (LLPs), are distinctive sequences that were initially identified by sequence scanning as having extraordinarily high hydrophobic moments (Figure 2) [38]. Subsequent studies on peptide analogs of these domains demonstrated high levels of structural similarity with naturally occurring cytolytic peptides [39], as well as the ability of the peptides to alter cell membrane permeability [40]. Peptide analogs of these domains have been demonstrated by circular dichroism spectroscopy to be generally unstructured in aqueous buffer, but to rapidly adopt an alpha-helical structure in membrane lipid or membrane-mimetic environments [41,42,43,44]. Additionally, the LLP domains demonstrate remarkable conservation of physicochemical properties, arginine residues, and secondary structures in spite of substantial sequence diversity [44]. Finally, a very recent structural study of LLP2 peptides interacting with fully hydrated lipid membranes provides the first atomic-level insight into CTT-lipid interactions. LLP2 peptides were demonstrated to interact with and to embed into the interface of membranes whose lipid content mimicked that of the T-cell membrane, but the same LLP2 peptides interacted weakly with and did not insert into membranes whose lipid content mimicked the viral envelope [45].

#### 2.3.2. CTT Membrane-interactive Domains are Conserved

The conservation of the physicochemical properties of the CTT is particularly interesting for the LLP regions in light of their proposed membrane associating characteristics [41,42,43,46], where the hydrophobic moment and charge may complement one another. The hydrophobic moment has been proposed as a measure of the tendency of a sequence to prefer the chemically-complex interfacial boundary between the hydrocarbon membrane interior and the aqueous phase [47]. The preference for the chemically complex membrane-water interface is suggestive of a role for the LLP regions as membrane-anchoring sequences. The insertion of the LLP sequences into the interfacial region would have a physical influence on the local membrane environment through the introduction of local curvature stress, as well as by affecting the lateral pressure profile in the membrane interior [48,49,50,51,52,53]. These physical alterations of the membrane environment by membrane-interacting LLP peptides may provide an explanation for the modulation of Env ectodomain conformation induced by point mutations in the LLP [54]. For example, Kalia *et al*. reported that the mutation of two highly conserved arginine residues to glutamates in LLP1 or LLP2 markedly altered the overall Env conformation, antigenic and functional properties. While the Arg to Glu mutations introduced by Kalia *et al*. maintain the amphipathic potential of the LLP2 region, they alter the net charge of the region from +3 to −1 [54]. The resulting overall negative charge of the region may impact its association with the negatively charged inner membrane leaflet by introducing charge-charge incompatibilities with the negatively charged phosphatidylserine headgroups. Differential association of LLP2 with the inner leaflet of the membrane would lead to a change in the local lateral pressure profile of the membrane, which, in turn, could lead to conformational changes in the protein by altering the arrangement of the transmembrane helices [49,51,53,55,56].

Recent experimental evidence supports this idea of differential membrane association, where Tristram-Nagle and colleagues determined the structure of wild-type (WT) and the previously characterized Arg to Glu mutant LLP2 peptides interacting with fully-hydrated lipid bilayers [45]. Their results demonstrated that the WT and mutant sequences associated and inserted differently into lipid membranes mimicking the composition of a T-cell membrane, with the Arg to Glu mutant sequence inserting ~15 angstroms (Å) more deeply into the hydrophobic core of the membrane, while the WT sequence was localized to the membrane-water interfacial region [45]. This study supports the concept that minor mutations in the LLP2 region can have a measurable impact on CTT-lipid association. Interestingly, the CTT has recently been demonstrated to also modulate the conformation of gp120 and the gp41 ectodomain in virus particles, where Env with a CTT deletion displayed a more open state, similar to the CD4-bound state [57,58]. This suggests the possibility that the CTT, and the LLP regions, in particular, evolves under pressure to maintain the “native” conformation of Env by maintaining specific, consistent interactions with the lipid membrane.

The observed conservation of arginine residues in the CTT supports the demonstrated unique functionality for the CTT. Briefly, studies demonstrating a transient exposure of LLP2 sequences during the cell-cell fusion process are suggestive of a mechanism to prefer arginine conservation relative to replacement with lysine [59]. Arginine-rich peptides have been demonstrated to cross cellular membranes, while peptides with lysine instead of arginine do not [60,61,62]. In addition, arginine-rich peptides have been demonstrated to deliver soluble proteins into the cytoplasm of live cells [63]. The observed apparent traversing of the membrane by LLP2 during the fusion process would seem, then, to require the presence of arginine and limit substitutions by lysine.

#### 2.3.3. CTT Function

In addition to the membrane lipid bilayer, the CTT has been shown to interact physically or functionally with other viral and cellular proteins. These functional interactions have been found to regulate: (i) Env incorporation into viral particles [64,65,66,67,68,69]; (ii) virion maturation [57,66,70,71]; (iii) overall Env conformation and functional properties [54,57,58]; (iv) endocytosis of Env from the cell surface to late endosomes [72,73]; and (v) viral transcription and replication. 

##### 2.3.3.1. Virion Env Incorporation

Seminal studies by Freed and Martin demonstrated that the CTT was implicated in the incorporation of Env into virions through interactions with the matrix (MA) domain of Gag. The initial study used site-directed mutagenesis to demonstrate that mutations in specific amino acids in Gag MA resulted in deficiencies in the incorporation of Env with a full-length CTT [64]. The MA mutations did not, however, lead to reduced incorporation of heterologous retroviral Env proteins with naturally short CTT sequences. Further, specific truncation of CTT sequences to seven or 47 residues (from the original 150) reversed the Env-incorporation block imposed by the MA mutations. This study demonstrated, for the first time, conclusive evidence of a functional role for the CTT through direct or indirect interactions with an intracellular partner, Gag MA. A later study provided evidence for a direct interaction between MA and the CTT [74]. 

The role and localization of sequences important in the MA-CTT interaction has been further elucidated. A subsequent paper by the same authors demonstrated that truncating up to 56 residues from the CTT (leaving a 94-residue CTT) resulted in no Env incorporation in viruses containing MA mutations, leading to deficiencies in Env incorporation, but that truncations of 93 amino acids or greater (leaving only a 57-amino acid CTT) relieved the block caused by MA mutations, resulting in efficient Env incorporation [65]. Later papers from this group localized the MA-CTT interaction to LLP2 in the CTT [68] and further demonstrated that the interaction was crucial for Env virion incorporation in a cell type-specific manner [69]. 

The CTT also appears to affect virion Env incorporation through interactions with the viral accessory protein, Nef. Functional interactions between Nef and the CTT dileucine motif also increase Env expression at the cell surface and virion incorporation [75]. This may be due to increased colocalization between Gag and Env in late endosomes, as shown for retroviral envelopes [76].

##### 2.3.3.2. Virion Maturation

Aiken and colleagues have further elucidated the interaction of the Env CTT with MA by examining the effect of pelleting immature HIV virus particles through detergent [77]. Pelleting particles through detergent strips away the viral lipid membrane in which the CTT is embedded, leaving viral protein cores. If the CTT and Pr55Gag interact, then gp41 should be associated with the pelleted viral cores. The results from this study demonstrated that the pelleted cores retained the major fraction of the gp41 found in untreated virions [77]. In contrast, gp41 did not remain associated with pelleted cores produced by similar detergent treatment of virions containing a truncated CTT [77]. Interestingly, a previous study using mature viral particles for a similar analysis demonstrated no association of the CTT with MA [78]. Thus, taken together, these results suggested that the association of the CTT with Pr55Gag was dependent on the maturation state of the virion.

This maturation-dependent association of the CTT with Gag has been extended to examine the role of the CTT in viral fusion. It is well-established that HIV particles are not infectious until proteolytic cleavage of the Pr55Gag into its constituent domains (MA, CA and NC, predominantly). Using a reporter assay to measure virus-cell fusion, Wyma *et al*. demonstrate that immature virions are less fusogenic than mature virions in a manner that is dependent on the CTT, as truncations of the CTT resulted in identical fusogenicity of immature and mature viral particles [71]. More recent results suggest that the extreme *C*-terminus of the CTT, LLP1, modulates the maturation dependence of infectivity, while the deletion of this region does not affect the incorporation of Env into immature viral particles [66]. The CTT has also been shown to modulate the mechanical stability of immature virus particles relative to mature virus particles, presumably through its interactions with Pr55Gag [70]. These results collectively demonstrate that the interaction of the CTT and Gag have specific effects on the infectivity and structural stability of HIV particles.

##### 2.3.3.3. Env Endocytosis

The gp41 CTT of both SIV and HIV have long been known to contain endocytic signals. The first was demonstrated in SIV, where a consensus YXXΦ motif in the CTT was shown to interact with members of the adaptor protein medium chain family [73]. It has also been demonstrated that CTT sequences interact specifically with the AP-2 clathrin adaptor [79]. Most recently, Byland *et al*. demonstrated that the CTT contains two functional endocytic signals: a 711GYXXΦ motif located near the *N*-terminus of the CTT and a dileucine motif at the extreme *C*-terminus [72]. Their results indicate that in order to completely abolish endocytosis of Env from the cell surface to late endosomes, both motifs must be mutated, suggesting that both the *N*- and *C*-termini of the CTT are cytoplasmically localized to interact with the cellular endocytic machinery.

##### 2.3.3.4. Transcriptional Regulation and Virus Replication

The CTT interacts with numerous cellular proteins (reviewed in [8,9,10]), and some of these associations function to modulate HIV-1 replication. Among the first cellular partners identified for the CTT was calmodulin. This interaction is associated with a decrease in the cellular protein synthesis activity [80,81]. Calmodulin, as a regulator of intracellular calcium concentration, regulates a variety of cellular enzymes and pathways, which has many potential outcomes on viral replication. The CTT has also been demonstrated to interact with p115-RhoGEF, a guanine nucleotide exchange factor (GEF) and activator of RhoA GTPase [82]. This functional interaction positively regulates HIV-1 replication in human T-cells, possibly by relieving RhoA inhibition of HIV-1 gene expression, suggesting that gp41 counteracts the inhibition applied by p115/RhoA to maintain viral replication [82,83]. The CTT also interacts with the cellular protein, Luman, a transcription factor belonging to the CREB/ATF family, destabilizing the full-length precursor of the active form of Luman, thus counteracting its repressive effect on HIV-1 long terminal repeat (LTR) activation and relieving Gag and Env production [84]. More recently, the interaction between the CTT and the cellular prohibitins has been shown to contribute to virus replication in a cell type-dependent manner [85]. Prohibitins are expressed in many cellular compartments and are involved in multiple functions, such as mitochondrial functions or transduction pathways [86,87]. However, how their interaction with the CTT supports HIV-1 replication in certain cell types remains to be determined. Finally, the CTT also activates the NF-κB canonical pathway through interaction with TAK1 [88]. As the HIV-1 LTR contains NF-κB binding sites, these observations suggest a function for this interaction in regulating HIV-1 gene expression. The importance of this activation was revealed in conditions of sub-optimally activated T-lymphocytes in which a mutated envelope that could not interact with TAK1 affected virus replication [88].

##### 2.3.3.5. CTT Influences Env Structure

One of the earliest observations that the CTT could influence the overall Env structure was provided by the insight that viruses with CTT-deleted Env proteins could infect target cells in a CD4-independent manner [89]. Until that time, the paradigm for HIV infection of target cells was that gp120 binding to CD4 was necessary to induce conformational changes that allowed binding to the coreceptor. Edinger *et al*. demonstrated that viruses with a CTT-deleted Env were able to infect CD4-negative, coreceptor-positive cells, suggesting a distinct conformation for Env that was dependent on the CTT.

Initially, direct evidence for CTT-dependent alterations in the Env structure was provided by differential reactivity between CTT-deleted and wild-type Env with conformationally-dependent antibodies. Edwards *et al*. demonstrated that truncation of the CTT to 27 amino acids resulted in increased binding by monoclonal antibodies directed to both the CD4 binding site and the CD4^−^ induced coreceptor binding site [90]. In Env with a full-length CTT, monoclonal antibodies directed to the coreceptor binding site only bound if the Env was preincubated with soluble CD4. The study also demonstrated the differential reactivity of conformational monoclonal antibodies directed at the ectodomain of gp41 between CTT-truncated and full-length CTT [90]. This study was the first to demonstrate that the CTT modulates the conformation of the overall Env structure, even in the non-covalently attached gp120, which is located on the opposite side of the membrane.

Later studies demonstrated that point mutations in the CTT could exert similar conformational effects as CTT truncation. Kalia *et al*. demonstrated that alterations in the CTT could modulate the antigenic conformation of both the gp120 protein and the gp41 ectodomain. Instead of utilizing large deletions in the CTT, however, Kalia *et al*. demonstrated that the mutation of two conserved arginine residues in LLP2 to glutamate was sufficient to alter the conformation of both gp120 and the gp41 ectodomain on the surface of Env-expressing cells [54]. The mutations were previously shown to have no effect on the levels of virion Env incorporation or viral replication, but decreased the efficiency of cell-cell fusion [91]. In addition to demonstrating antigenic distinctions between wild-type and mutant Env on the cell surface, differences were seen in the viral sensitivity to antibody-mediated neutralization by antibodies directed at the CD4 binding site, with the mutant virus demonstrating an approximately 40-fold decrease in neutralization sensitivity [54]. This study demonstrated that point mutations in the CTT were sufficient to alter overall Env conformation and, equally importantly, provided the first pieces of information regarding the critical nature of the conserved arginine residues in the LLP regions.

A recent study has examined the effect of CTT-dependent alterations on Env antigenicity on the surface of viral particles [57]. Joyner *et al*. demonstrated that Env on immature virions (protease deleted) reacted differently to a number of conformationally-dependent monoclonal antibodies in a manner that was dependent on the presence or absence of the CTT [57]. This study provided the first direct evidence that the CTT plays a major role in modulating the conformation of the CTT in the virion in addition to on the cell surface.

There is also recent structural evidence for the role of the CTT in the modulation of overall Env conformation. Subramaniam and colleagues determined the cryo-EM structures of CTT-deleted Env on the surface of SIV viral particles [58]. In comparison to wild-type virus containing the full-length CTT, CTT-deleted Env existed in a naturally “open” state, displaying large differences in the localization of electron density, which are consistent with the conformational changes normally associated with CD4 binding. This study, finally, provides the first conclusive structural evidence that the CTT sequences can, in addition to their functional roles, also serve to modulate the overall Env structure and function.

## 3. Conclusions

The *C*-terminal domains of varying retroviruses, which, at first glance, appear to have little in common with each other, aside from their position in the Env protein sequence, display some remarkably similar properties and functions. While the *C*-terminal domains from each protein have some very distinct functions, it is interesting to note that in all three proteins discussed here, the *C*-terminal domain has been experimentally implicated in the regulation of viral fusogenicity through the modulation of ectodomain TM conformation. While there is no sequence homology between these viral *C*-terminal domains and, thus, likely little structural similarity, this apparent functional homology serves as a striking reminder of the evolutionary relationship amongst this family of viruses. Perhaps it is important to consider, based on the comparisons and resulting similarities presented here for a single (small) domain of one of the essential genes (Env) from three retroviruses distinct from each other in sequence, species tropism and disease, that the retroviral research fields might still have much to learn from each other. 
